# Apomixis and strategies to induce apomixis to preserve hybrid vigor for multiple generations

**DOI:** 10.1080/21645698.2020.1808423

**Published:** 2020-09-02

**Authors:** Sajid Fiaz, Xiukang Wang, Afifa Younas, Badr Alharthi, Adeel Riaz, Habib Ali

**Affiliations:** a Department of Plant Breeding and Genetics, The University of Haripur 22620, Khyber Pakhtunkhwa, Pakistan; b College of Life Sciences, Yan’an University, Yan’an, Shaanxi, China; c Department of Botany, Lahore College for Women University, Lahore, Pakistan; d College of Science and Engineering, Flinders University, Adelaide, Australia; e University College of Khurma, Taif University, Taif, Saudi Arabia; f Biotechnology Research Institute, Chinese Academy of Agricultural Sciences, Beijing, China; g Department of Agricultural Engineering, Khawaja Fareed University of Engineering and Information Technology, Rahim Yar Khan, Pakistan; h Department of Entomology, Sub-Campus Depalpur, University of Agriculture Faisalabad, Faisalabad, Pakistan

**Keywords:** Hybrid vigor, flowering plants, apomixis, CRISPR/CAS system

## Abstract

Hybrid seeds of several important crops with supreme qualities including yield, biotic and abiotic stress tolerance have been cultivated for decades. Thus far, a major challenge with hybrid seeds is that they do not have the ability to produce plants with the same qualities over subsequent generations. Apomixis, an asexual mode of reproduction by avoiding meiosis, exists naturally in flowering plants, and ultimately leads to seed production. Apomixis has the potential to preserve hybrid vigor for multiple generations in economically important plant genotypes. The evolution and genetics of asexual seed production are unclear, and much more effort will be required to determine the genetic architecture of this phenomenon. To fix hybrid vigor, synthetic apomixis has been suggested. The development of *MiMe* (mitosis instead of meiosis) genotypes has been utilized for clonal gamete production. However, the identification and parental origin of genes responsible for synthetic apomixis are little known and need further clarification. Genome modifications utilizing genome editing technologies (GETs), such as clustered regularly interspaced short palindromic repeats (CRISPR)/CRISPR-associated protein (cas), a reverse genetics tool, have paved the way toward the utilization of emerging technologies in plant molecular biology. Over the last decade, several genes in important crops have been successfully edited. The vast availability of GETs has made functional genomics studies easy to conduct in crops important for food security. Disruption in the expression of genes specific to egg cell *MATRILINEAL* (*MTL*) through the CRISPR/Cas genome editing system promotes the induction of haploid seed, whereas triple knockout of the *Baby Boom* (*BBM*) genes *BBM1, BBM2*, and *BBM3* cause embryo arrest and abortion, which can be fully rescued by male-transmitted *BBM1*. The establishment of synthetic apomixis by engineering the *MiMe* genotype by genome editing of *BBM1* expression or disruption of *MTL* leads to clonal seed production and heritability for multiple generations. In the present review, we discuss current developments related to the use of CRISPR/Cas technology in plants and the possibility of promoting apomixis in crops to preserve hybrid vigor. In addition, genetics, evolution, epigenetic modifications, and strategies for *MiMe* genotype development are discussed in detail.

## 1. Introduction

**Figure 1.**
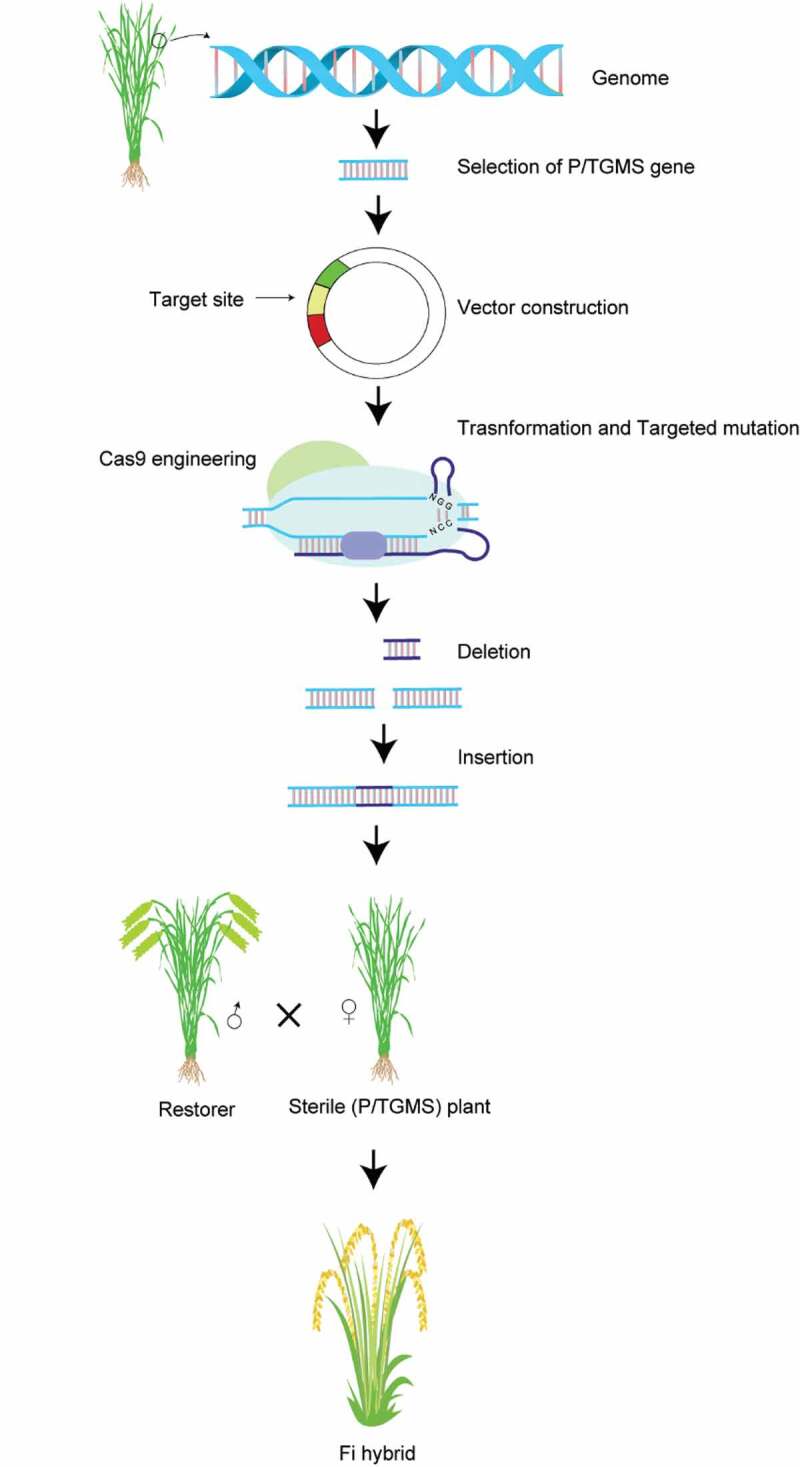
Schematic description of the hybrid development utilizing CRISPR/Cas genome editing system. The two line hybrid development through knockout of P/TGMS = photoperiod/thermo-sensitive genic male sterile genes from elite cultivar. The selection of target in any exon of gene under consideration and later targeted modification through CRISPR/Cas technology helps to induce mutations. The frame shift mutation (Deletion and Insertion) is mostly desirable. The screening of mutants to get male sterile plants, crossing with compatible restorer generate F_1_ hybrid.

Scientific, logistical, and humanitarian approaches are simultaneously involved to ensure food security, starting with farmers and breeders and further extending to policymakers and governments. A universal solution for sustainable food security is difficult owing to differences in cultural values and geographical regions, environments, and technologies. Although these challenges are substantial, there is great potential to increase the efficiency and productivity of current agricultural products. The powerful breeding techniques that we have at our disposal are being used to exploit heterosis in commercially important crops. Hybrid seed production is a successful technology, and hybrid seeds produced either by three-line or two-line systems to achieve supreme qualities, i.e., premium yield and quality along with resistance against biotic and abiotic stresses, have been cultivated for a long time.^1^ Among the two-line and three-line hybrid development systems, the former is more advantageous than later because in this system, photoperiod-dependent/thermogenic male sterile lines have been successfully developed. Moreover, the application of CRISPR has further reduced the time required for hybrid seed development.^2^ Recently, a novel male fertility-related gene in wheat, *Ms1*, was identified, and the biallelic frameshift mutation resulted in complete male sterility that can be utilized for commercial hybrid seed production.^3^ Furthermore, the knockout of *ZmTMS5* and *OsTMS5* in maize and rice, respectively, resulted in thermosensitive lines. These lines contain fertile female parts and have been successfully utilized for hybrid seed production.^4^ Moreover, a transgene-free maize male sterile line was developed through the targeted mutagenesis of the *MS8* gene and proved to be a valuable source for hybrid development^5^ (Fig. 1). However, a major challenge with hybrid crops thus far has been that unlike other crops, their progeny segregate, and the next generation is unable to maintain heterosis.^6^ These limitations of sexual reproduction make apomixis a feasible strategy for preserving hybrid seed qualities for multiple generations.^7^

Apomixis exists naturally and produces offspring that are genetically identical to the mother plant. Naturally, the phenomena of apomixis are widespread but exceptional. In 400 families of flowering plants, approximately 10% undergo the phenomenon of apomixis, which constitutes only 1% of the 40,000 species in those families.^8^ Under natural conditions, multiple developmental pathways controlled by various molecular mechanisms play an integral role in achieving apomixis.^9^ Apomictic pathways have been categorized as examples of adventitious embryony, a sporophytic type of apomixis, while megaspore mother cells (diplospory) and nearby nucellar cells (apospory) exhibit two gametophytic forms of apomixis. In gametophytic apomixis, a chromosomally unreduced embryo sac develops from diplospory or from apospory in a process termed apomeiosis.^10^ Parthenogenesis, the development of the unreduced, unfertilized egg into an embryo, constitutes the second step of the apomictic process.^11^

Synthetic apomixis has been proposed by researchers to overcome the genetic segregation of F_1_ hybrids of different crops. Genetic analysis of the inheritance of apomixis in different plant species, cereals, grasses, and related genera, i.e., *Tripsacum, Pennisetum, Panicum, Bracchiaria*, and *Paspalum*, detected a single chromosome segment responsible for inducing apomixis.^12^ Nevertheless, efforts to transfer chromosomal segments responsible for the promotion of apomixis remain elusive owing to genetic evolution load.^11^ In addition, induced mutations cause complete male or female sterility and seldom produce unreduced gametes.^13^ However, the molecular mechanisms controlling apomixis for clonal seed formation are largely unknown, and novel plant breeding strategies are desirable to unearth the genetic mechanism controlling such interesting phenomena. If synthetic apomixis is introduced in any crop vital for food security, it can help fix and propagate genotypes regardless of the genetic complexity in controlling a particular phenotype, ultimately enhancing the application of heterosis fixation in agriculture.^14^ Because of these enormous prospects, ingress of apomixis in commercially important crops is considered a hotspot to be studied and explored by plant biologists and the seed industry ^15,16^ introduced synthetic apomixis in a few fruit crops, i.e., citrus and apple; however, epigenetic modifications involve severe seed abortion, leading to the failure to transfer apomixis in major crops.^10^ Recent studies have discussed genetic and biotechnological approaches,^17^ integration of knowledge from various aspects of plant breeding,^18^ and methods for exploiting genome editing, especially the CRISPR/Cas genome editing system, for improving yield and preservation of heterosis in hybrids.^19,20^ The scientific community, equipped with genome editing expertise, has played a significant role by transferring relevant and reliable information to beginners in genome editing, in contrast to the proprietary nature of methods utilizing zinc-finger nucleases (ZFNs). In addition, several online platforms are now freely accessible to assist researchers with their efforts related to genome editing, especially using CRISPR.^21^ Here, in the present review, we discussed details of apomixis along with the utilization of the CRISPR/Cas genome editing system to develop synthetic apomixis as an alternative method to preserve hybrid seed vigor in economically important crops.

## 2. Evolution of Apomixis

**Figure 2.**
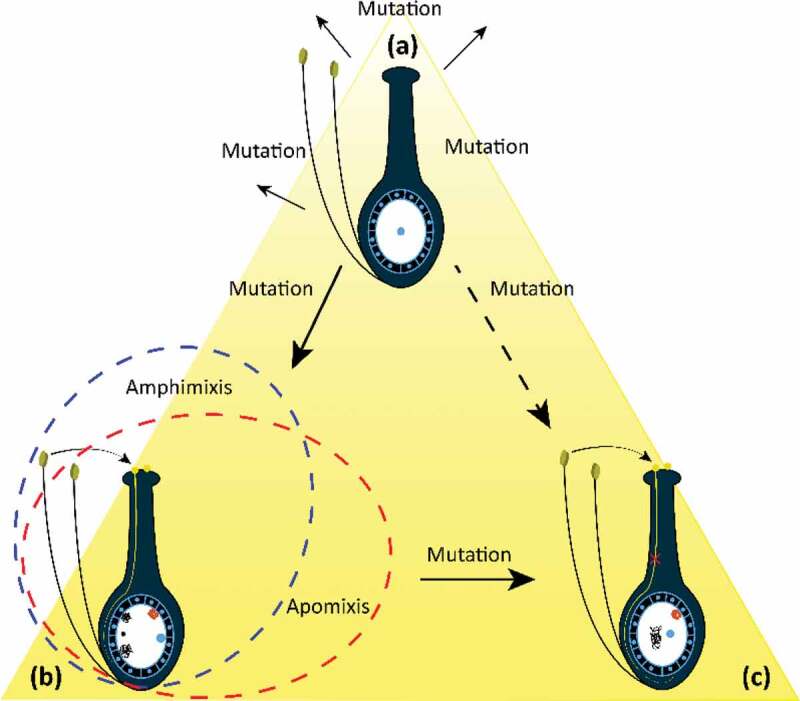
Illustration of the emergence of apomictic types in plants modified from Fei et al. (2019).^30^ (a) Amphimixis, (b) facultative apomixis, and (c) obligate apomixis.

Apomixis is an interesting plant attribute that allows maternal clones via seed production. Apomixis is an intangible but revolutionary characteristic used in plant breeding and for preserving hybrid seed qualities. Recent findings lead to the conclusion that apomicts are useful, with the potential to develop more research interest in the evolutionary process of asexual seed production in flowering plants. It is believed among researchers that apomixis alone has the ability to revolutionize twenty-first century agriculture in both developed and developing countries. Apomixis has been reported in approximately 35 angiosperm families and in more than 300 plant species.^22^ Unfortunately, except for a few forage grasses and fruit trees, apomixis phenomena are not obvious in crop species. It is commonly believed that apomixis phenomena mostly occur in polyploid genotypes^23^; however, the discovery of apomixis in diploid species^24^ abolished the notion that polyploidy was necessary for apomixis.^25^ Four perennial *Cenchrus* spp. were found to exhibit natural apomixis, i.e., *C. myosuroides* is an obligate sexual, diploid apomictic, and *C. setigerus, C. echinatus*, and *C. myosuroides* are polyploid and sexual in nature, indicating apomixis without polyploidy. Thus, there is still a quest for in-depth research to challenge the strong but less understood correlation between polyploidy and apomixis. Moreover, the reliable transmission of maternal genotypes to the next generations also needs sufficient scientific investigation due to genetic variability among apomictic populations. There are at least three proposed mechanisms that may cause genetic variability in apomictic plants or species.^26^ First, new mutations accumulate^22^; second, irregular sexual reproduction causes recombinant genotypes in populations, a phenomenon often called leaky apomixis^27^; and third, facultative reproduction occurs.^28^ Many researchers around the globe have analyzed and discussed these mechanisms in detail^29^ (Fig. 2).

## 3. Genetic Basis and Molecular Control of Apomixis

Apomixis is qualitative in nature and involves components of genetic analysis. Molecular studies to unearth the genetic basis for apomixis are unable to explain mysterious phenomena because most apomicts are not important crops.^31^ There is a well-established association between polyploidy and gametophytic apomixis.^32^ The discoveries of apomictic phenomena in diploid plant populations disassociated the absolute necessity for polyploidy to occur along with apomixis.^25^ However, some researchers have suggested that the genetic control of apomixis is not an independent trait but is triggered by epigenetic temporal and spatial modifications of the sexual system.^33^ Earlier, the genetic control of apomixis was thought to be controlled by recessive genes, while their balance may change after each successful crossing. Recently, a new concept for the inheritance process predicted the involvement of a single major regulatory gene or a set of key dominant genes, allowing megaspore mother cells or somatic nuclear cells to form an embryo sac and an embryo from unreduced egg cells without fertilization.^,32,34^ However, the spatial and temporal distribution of these genes during developmental processes is still not well understood. The genetic mechanism of asexual reproduction is complicated in nature; therefore, interspecific hybrids or intraspecific hybrids within agamic complexes are widely studied in the context of the segregation (apomeiosis, parthenogenesis, and functional endosperm development) of apomixis. Moreover, the phenotypic analysis of plant generation reproduction either asexually or sexually involves time-consuming cytology or progeny testing. A single gene with regulatory function was initially proposed to promote asexual reproduction. Recently ^31^ reported that three key components (apomeiotic megaspores, parthenogenic-unreduced egg cells, and modified endosperms) of gametophytic apomixis depend on three independent Mendelian loci of major influence. The relationship between rich retrotransposon regions of heterochromatin and apomictic mechanisms has elevated the possibility for the role of DNA and/or RNA interference in regulating the expression of apomictic genes.^35^ Some researchers have reported the influence of specific genes encoding novel proteins for new functions that are absent from plants with sexual reproduction and ultimately the identification of candidate genes/loci. Some genes originally fine mapped and characterized in or from species that reproduce sexually may play an important role in promoting apomixis through loss of function or change in function of genes, differentiating between apomictic and sexual pathways. However, the genetic factors controlling apomixis are also associated with some lethal genes influencing the normal functioning of both male and female gametes, which needs further investigation.

Apomictic species exhibit suppressed recombination; however, the genes controlling the various components of apomixis have been identified, and sequencing of these loci has revealed a number of genes with the potential to have critical roles in apomixis. Random amplified polymorphic DNA-based analysis helped in the identification of the *apospory-specific genomic region* (*ASGR*) of *Pennisetum* in both sexual and apomictic plants.^36^ *ASGR* sequences revealed that *ASGR* contains both gene-rich and gene-poor segments, several genes that may play a role in apomictic development, and many classes of transposable elements, and *ASGR* does not exhibit large-scale synteny with either rice or sorghum genomes but does contain multiple regions of microsynteny with these species.^37^ The bacterial artificial chromosome (BAC) clone sequencing of *ASGR* from *Pennisetum* and *Cenchrus* revealed 40 putative protein-coding regions, two of which showed sequence similarity to the *BABY BOOM* (*BBM*) gene. The *BBM* gene was identified in *Brassica napus* as an AP2-domain transcription factor, and overexpression analysis in *Arabidopsis* showed its involvement in embryo development from vegetative tissues.^38^ The *ASGR-BBM* possesses potential and is a candidate gene for the induction and/or maintenance of apomictic events.^37^ Amplified fragment length polymorphism (AFLP) marker screening in apomictic and sexual plants of *Hypericum* led to the identification of the locus *HYPERICUM APOSPORY*. The perfect co-segregation of the AFLP was further utilized to screen the BAC library of *Hypericum*. The screening helped in the identification of a single clone containing aubiquitin-mediated E3 ligase.^39^ This *ARIADNE 7*-like E3 ligase (*HpARI*) has been proposed as a candidate for the *HAPPY* locus, as one of the four *HpARI* alleles within the tetraploid apomict is truncated compared to that in sexual plants. *HpARI* acts as a negative regulator and interacts with the remaining three alleles. The E3 ligase is responsible for ubiquitin-mediated protein degradation involved in embryo sac development. With roles in mitotic spindle function and nuclear fate in the developing maize embryo sac, the MATH-BTB protein MAB interacts with an E3 ubiquitin ligase component (Cullin3a).^40^ Any modification in the expression or function of the *HpARI* E3 ligase may impact embryo sac development and apomixis in *Hypericum*. Spatial and temporal alterations in the expression of sexual pathway-controlling genes may cause apomixis; therefore, comparative gene expression analysis may provide an opportunity to identify such genes. The low depth express sequence tag (EST) libraries consisting of the apomictic and sexually propagated aposporous grass *Brachiaria brizantha* revealed sequence similarity among genes involved in female embryo sac development.^41^ Characterization of identified genes mainly expressed in aposporous initial cells was performed. The helicase *BbrizHelic, BbrizAGL6* transcription factor, and *BbrizSti1* stress-inducing protein were found to play integral roles in aposporous initial cells.^42^ Apomixis-associated *APOLLO* locus is suggested to be one of the important apomixis-related genes in *Boechera. APOLLO* transcripts are down-regulated in sexual ovules when they enter meiosis and are up-regulated in apomeiotic ovules at the same stage of development.^43^ *APOLLO* has both ‘apoalleles’ and ‘sexalleles,’ implying apomixis-associated polymorphisms. In *Poa pratensis*, cDNA-AFLP helped in the identiﬁcation and characterization of two candidate genes: *SOMATIC EMBRYOGENESIS RECEPTOR-LIKE KINASE* (*PpSERK*) and *APOSTART*.^44^ Both the *PpSERK* and *APOSTART* genes were isolated in two copies, and there were several alleles present within the *P. pratensis* genome. The *PpSERK* gene is a tyrosine kinase that switches aposporous initial cells to form and develop embryo sacs in somatic cells.^44^ *APOSTART* contains a lipid-binding START domain and is believed to have a role in meiosis. *APOSTART* may also be related to programmed cell death and the degeneration of nonfunctional megaspores, with one of the two isolated copies (*APOSTART1*) overexpressed in sexual lines relative to those in apomicts. The *Arabidopsis APOSTART1* ortholog is expressed in mature female embryo sacs and developing embryos, and the phenotype of *APOSTART1/APOSTART2* double mutants suggests that this gene has a role in embryo and seed development.^11^ Several other genes identified by several researchers play important roles in controlling asexual reproduction and can be utilized in the future to understand their regulatory functions (Table 1).

**Table 1.** Apomixis-related candidate genes and related information.

Genes	Function/Process involved	Description	Species	References
*ASGR–BBML*	Apospory	Multiple copies of the *PsASGRBABY BOOM-like* (*PsASGRBBML*) gene reside within the apospory-specific genomic region	*Pennisetum squamulatum*	^9^
*LOSS OF APOMEIOSIS (LOA)*	Apospory	Responsible for apospory	*Hieracium Praealtum*	^45^
*Deficiens*	Apospory	Deficiens is a member of the B class of floral homeotic regulators that have been shown to specify petal and stamen identity according to the ABC model of floral development	*Hieracium piloselloides*	^46^
*PnTgs1-like*	Apospory	PnTgs1-like (formerly N69) encodes a trimethylguanosine synthase-like protein whose function in mammals and yeast is critical for development, including reproduction	Paspalum notatum	^47^
*GID1*	Apospory	Gibberellin-insensitive DWARF1	*Brachiaria brizantha*	^48^
*MSP1*	Adventitious embryony	Multiple sporocyte controls early sporogenic development	*Oryza sativa*	^49^
*RWP*	Adventitious embryony	RWP is the key gene controlling polyembryony	*Citrus reticulate*	^14^
*SERK*	Adventitious embryony	Somatic embryogenesis receptor-like kinase	*Poa pratensis*/*Paspalum notatum*	^50^
*ORC*	Adventitious embryony	ORC (ORIGIN RECOGNITION COMPLEX) is a multiprotein complex which controls DNA replication and cell differentiation in eukaryotes	*Paspalum simplex*	^51^
*FIE*	Autonomous endosperm	Fertilization independent endosperm	*Malus hupehensis*/*Solanum lycopersicum*	^52^
*DYAD/SWITCH1*	Diplospory	Result in defects in female meiosis/affect meiosis in megasporocytes	*Arabidopsis, dyad* mutant	^53^
*Apostart*	Diplospory	Speculated to participate in the formation of 2 n eggs in apomixis	*Medicago sativa*	^44^
*DMC1*	Diplospory	Participation in meiosis	*Arabidopsis*	^54^
*Argonaute 9 (AGO 9)*	Diplospory	*AGO9* controls female gamete formation by limiting the specification of gametophyte precursors in a non-cell autonomous manner	*Arabidopsis, AGO9 mutant*	^55^
*AGO 104*	Diplospory	*AGO 104* and *AGO9* are homologous genes, and their defects can produce a phenotype similar to apomixis, giving rise to up to 70% of functional unreduced female gametes	*Tripsacum*	^56^
*AGAMOUS-LIKE 62*	Inhibit endosperm cellularization	Agamous-like 62 (AGL62) is a member of the MADS-box gene, an increase in its expression level can inhibit the development of the germ and inhibit endosperm cellularization	*Arabidopsis, fis2* mutant	^57^
*lorelei-like/n20gap-1*	Might play a role in the final stages of the apomixis developmental cascade.	Encodes a GPI-anchored protein previously associated with apomixis.	*Paspalum notatum*	^58^
*dmt*	May be involved in differentiation between apomictic and sexual reproduction	The expression of the dmt (DNA methyl transferases) gene was significantly different between sexual and apomictic reproduction. The absence of dmt102 and dmt103 can produce a phenotype similar to apomixis	maize-Tripsacum hybrid	^59^
*APOLLO*	Apomixis-associated polymorphisms	Has ‘apoalleles’ and ‘sexalleles’, apomictic *Boechera* spp. are heterozygous for the *APOLLO* gene, sexual genotypes are homozygous for sex alleles	*Boechera retrofracta*	^43^
*DEMETER (DME)*	DNA demethylase	Component of PRC2, in mutants autonomous endosperm forms. Responsible for endosperm maternalallele-speciﬁc hypomethylation at the MEDEA (MEA), regulation of gene expression by genetic imprinting	*Arabidopsis*	^60^

## 4. Epigenetic Control of Apomixis

The variable developmental processes and cells involved in apomictic mechanisms indicate the influence of epigenetic regulatory mechanisms in controlling apomictic processes. To investigate the hypothesis of cytosine methylation histone modification and comparative analysis of small-RNA have been undertaken both in apomictic and sexually propagated plants. In higher plants, the activation or movement of transposable elements (TEs) covers a significant proportion of the genome, influencing the evolution of the genome, alternation in gene expression, and frame shift mutations.^61^ To maintain the genomic integrity of the host genome, TE movement is important for regulation. The centromeric or telemetric regions are generally saturated with TEs with a high rate of methylation and packed as heterochromatic regions. In maize, it has been proven that in heterochromatin regions, TEs are specific for methylation, indicating their integral role in the alternation of structural and functional properties in plants. The role of retrotransposons in apomictic development has also been proposed; however, further proof of concept from both apomictic and sexually propagated plants is needed. From the *Cenchrus ciliaris* public database of the EST/BAC sequence, several classes of retrotransposons were identified, and their possible role in apomixis was elucidated. Based on expression analysis from 19 identified retrotransposons, six were found to be associated with apomictic activity. Among the six retrotransposons, C-105 was further utilized to understand epigenetic regulation based on its differential activity in both apomictic and sexually propagated plants of *C. ciliaris*. Bisulfite sequencing of retrotransposons (~0.3 kb fragment) showed 95% methylation of cytosine in sexually propagated plants compared to apomictic plants. The reversible ability of epigenetic modification in sexual pathways has also been hypothesized. The analysis of *Arabidopsis* mutant ovules epigenetically controlled by the sRNA-mediated silencing pathway involving the ARGONAUTE 9 (AGO9) protein showed a transition from apomictic to sexual reproduction.^49^ The loss of function of the mutant protein *ago104* homologue to maize AGO9 showed apomictic-like characteristics, with ~70% functional female gametes. The comparative analysis of sexually reproductive maize with its apomictic wild relative *Tripsacum* further supported the epigenetic regulation of apomixis. A small number of chromatin enzymes, e.g., CMT3 and DRM2, showed differences in expression among apomictic and sexual plants. Moreover, the involvement of mutant alleles of genes responsible for DNA methylation and siRNA pathways in apospory or diplospory of *Arabidopsis* and maize has been recently revealed. Moreover, epigenetic modiﬁcations in the apomictic hybrids and polyploids lead to a collapse of the timing of developmental pathways, where developmental steps that are normally expressed successively occur simultaneously causing apomeiotic development of the embryo sac and full expression of apomixis.^62^ *MEDEA* (*MEA*) gene is involved in the control of fertilization-independent development of endosperm. This gene is associated with the epigenetic regulatory polycomb repressive complex 2 (PRC2). The function of PRC2 is silencing of gene expression via trimethylation of histone H3 at lysine 27 (H3K27me3). Loss of function of the *MEA* in *Arabidopsis* mea mutants resulted in the development of seeds independent of fertilization in *Arabidopsis*,^63^ the trait that is observed in some apomicts. However, there is still a quest to understand the underlying mechanism linking epigenetic modification with the establishment of apomixis in plants. Thus, the underlying mechanism/theory controlling epigenetic regulation of apomixis is worth studying, especially with regard to its ability to facilitate reversion to sexual propagation from the apomictic mode.^22^ The genetics, genomics, and epigenetic modifications that promote apomixis in flowering plants are well studied, whereas studies on induced apomixis through genome editing techniques, i.e., the CRISPR/Cas system, are at the foundation stage, as only a few reports have been published. The reports indicated the potential role of engineered apomixis in preserving hybrid seed vigor, which will ultimately lower the cost of hybrid seeds that farmers must buy every year.

## 5. Synthetic Apomixis through the *MiMe* Strategy

There are three most common approaches in plant breeding to convert crop plants from sexual to apomictic modes of reproduction: i) wide crosses with apomictic wild relatives, ii) mutation breeding, and iii) genetic transformation techniques. Sexual hybridization to transfer apomixis through interspecific hybridization relies heavily on the availability of wild relatives. In maize, apomixis was transferred from its wild relative *Tripsacum* through hybridization, whereas the hybrid produced after a series of backcrosses was sterile in nature, and facultative apomicts were not produced to recover the maize genome.^64^ Thus far, it is generally believed that the transfer of apomixis through wide crosses is not a successful approach. Moreover, the application of artificial mutation studies has provided significant evidence regarding the genetic architecture of apomixis. Meiosis and mitosis are distinguished based on three integral characteristics. First, DNA double standard breaks (DSBs) are induced with recombination and pairing between homologous chromosomes. Second, the first cell division leads to monopolar orientation of kinetochores of sister chromatids. Third, after genome duplication, the division of cells takes place two times.^65^ The genetic factors controlling these three developmental processes have been identified in plants. The genes *SPO11-1, SPO11-2, HOMOLOGOUS PAIRING ABERRATION IN RICE MEIOSIS1 (PAIR1), PUTATIVE RECOMBINATION INITIATION DEFECT1 (PRD1), PUTATIVE RECOMBINATION INITIATION DEFECT2 (PRD2), MEIOTIC TOPOISOMERASE VIB-LIKE (MTOPVIB), DSB FORMATION (DFO), CENTRAL REGION COMPONENT 1 (CRC1*), and *P31*^comet^ are indispensable for the induction of DSBs in plants. The induced mutations in the abovementioned genes disrupt the normal pairing and recombination of homologous chromosomes.^66^ The monopolar orientation of kinetochores during the first meiotic division is mainly regulated by the meiotic cohesion complex controlled by a major component *REC8*.^67^ The genes *OMISSION OF SECOND DIVISION* (*OSD1*) and *TARDY ASYNCHRONOUS MEIOSIS* (*TAM*) control entry into the second division of meiosis. Mutations in any of these genes impact the second division, leading to the production of diploid gametes in both males and females.^68^ The *spo11-1* and *rec8* double mutant produced a mitotic-like ﬁrst meiotic division.^69^ The *osd1, rec8*, and *spo11-1* triple mutant and *tam, rec8* and *spo11-1* triple mutant exhibited apomeiosis phenotypes in which meiosis switched into mitosis-like division, and this genotype was called *MiMe*.^65^ The *MiMe* strategy helped in the development of viable diploid gametes and their successful transfer into rice by combining mutations in the genes (*PAIR1, REC8*, and *OSD1*) involved in the same process, suggesting that this strategy could be widely applied in ﬂowering plants.^8^ Synthetic apomixis can be achieved by eliminating one set of chromosomes from either parent that has to be eliminated. Centromeres are the loci where the microtubule of the spindle is located and are essential for chromosome segregation during cell division. Centromeres are speciﬁed by a centromere-speciﬁc histone 3 (CENH3) protein in plants. The engineered genome elimination line (GEM) can be achieved through the application of CENH3 and further crossed with wild-type plants. Clonal seeds were generated by crossing *MiMe* plants with the GEM line and ultimately opening a plethora of options for clonal reproduction through seeds with mechanisms similar to apomixis.^70,^^71^Nonomura et al. (2007) reported the ortholog *AGO5* in rice that proved to play an integral role in premeiotic mitosis and meiosis; similarly, in maize, the lack of the ortholog *AGO9* leads to the production of viable gametes without meiosis.^56^ In *Arabidopsis*, the screening of parallel mutants for apomixis helped in the identification of genes responsible for the initiation of fertilization-independent seed (*FIS*) development. These *FIS* genes encode proteins that resemble members of the polycomb-related complex.^72^ Mutants of *FIS* genes are known to trigger endosperm development through an asexual mode of reproduction to varying extents. However, embryo initiation for most *fis* mutants is either low or does not occur. However, the epistatic effect of genes conferring the natural process of seed development is believed to be a limitation to obtaining apomictic plants. To date, several genes and their mutants have been developed to promote apomixis in various plant species and are well reviewed by Hand and Koltunow (2014); Barcaccia and Albertini (2013).^10,11^ The genetic transformation approach contains much potential, but limited genetic information is the only limiting factor. Map-based cloning for genes controlling apomixis in crop plants is relatively slow owing to the suppressed recombinants and excess repeated DNA sequences in apomixis-associated genomic regions. However, in a few plant species, apomictic genes were detected utilizing deletion studies.^45^ Recently, another approach called “conditional apomixis” has gained scientific ground, **switching^73^ the default mode of the plant reproduction system to an apomictic or sexual mode of reproduction.^74^ Conditional apomixis in plants can be achieved utilizing special promoters whose expression can be altered by certain chemicals or through epigenetic factors.^35^ However, the classical methods in plant breeding are time-consuming, labor-intensive, costly, unreliable, and not flexible. Due to these limitations in classical breeding approaches, modern genome editing technologies have gained scientific ground and have been widely utilized by plant breeders to meet their objectives with more ease and precision.

## 6. Synthetic Apomixis through Genome Editing to Preserve Hybrid Vigor

Currently, CRISPR technology is widely utilized with tremendous success in agriculture, especially for crops essential for food security, i.e., rice, wheat, maize, *Arabidopsis*, cotton, tomato, and potato. The user friendliness and low cost of genome editing have spurred research on crop trait development not only in academia but also with private companies dealing with agricultural products.^75^ Moreover, genome editing technology has not only accelerated breeding efforts to improve yield, grain quality, resistance against abiotic and biotic stresses and domestication in plants but also to develop plants that can withstand diverse environments with high-value traits, such as hybrid vigor or heterosis, which has proven to be a major challenge for researchers and farmers. These are significantly desirable goals that could change the agricultural production system. De novo targeted modification can alter the sexual mode of reproduction to one of apomixis and has been successfully employed in *Arabidopsis*.^70^ The *BBM* gene belongs to the *APETALA 2/ETHYLENE RESPONSE FACTOR* (*AP2/ERF*) DNA-binding domain family and is expressed in sperm cells. The *AP2/ERF* gene family is divided into the *ERF-like* category, which has a single *AP2* domain, and the *AP2-like* category, which contains two *AP2* domains (repeats 1 and 2) that are similar to each other and separated by a linker region. In *Pennisetum squamulatum*, apomixis is transmitted by a physically large, hemizygous, non-recombining chromosomal region, the *ASGR*.^12^ Several copies of *PsASGR-BBML* genes are considered candidate genes promoting parthenogenesis due to their strong linkage between apomictic *Pennisetum*/*Cenchrus* species. ^37,76^ Conner et al. (2013) reported a mutant of the *CcASGR-BBML* gene in *Cenchrus ciliaris* that ultimately lost its ability to experience parthenogenesis; moreover, *ASGR-BBML* has also been found in *Arabidopsis* and *Brassica*., 38^,^^9^successfully reduced the expression of *PsASGR-BBML* in apomictic F1 RNAi transgenic plants and endorsed the key role of *PsASGR-BBML* in promoting parthenogenesis in *Pennisetum squamulatum*, which can further lead toward haploid induction to vigorously obtain homozygous transgenic lines for breeding.^7^ Khanday et al. (2019) reported that triple knockout of the genes *BBM1, BBM2*, and *BBM3* caused embryo arrest and abortion, phenotypes which were fully rescued by the male-transmitted *BBM1*. These findings suggest that the requirement for fertilization in embryogenesis is mediated by male genome transmission of pluripotency factors. When genome editing to substitute the mitosis for meiosis (*MiMe*) phenotype^7,48^ is combined with the expression of *BBM1* in the egg cell, clonal progeny can be obtained that retain genome-wide parental heterozygosity. The synthetic asexual-propagation trait is heritable through multiple generations of clones, also known as the clonal *fix* strategy. Moreover, Wang (2019)^14^ reported that multiplex editing of three (*REC8, PAIR1*, and *OSD1*) key meiotic genes in hybrid rice led to the production of clonal diploid gametes and tetraploid seeds. Next, editing of the *MTL* ^77,4^gene involved in fertilization resulted in the induction of haploid seeds in hybrid rice. By simultaneous editing of these four endogenous genes in hybrid rice using the CRISPR/Cas9 system, plants that are able to propagate clonally through seeds were obtained. The quadruple *aop* (Apomictic Offspring Producer) mutants obtained through the knockout of *OsSPO11‐1, OsREC8, OsOSD1*, and *OsMATL* produced the *MiMe* phenotype. These mutants have the ability to produce apomictic plants.^47^ However, thus far, the number of viable clone seeds with their original hybrid genetics intact has been much lower than expected, and this area needs further research.

**Figure 3.**
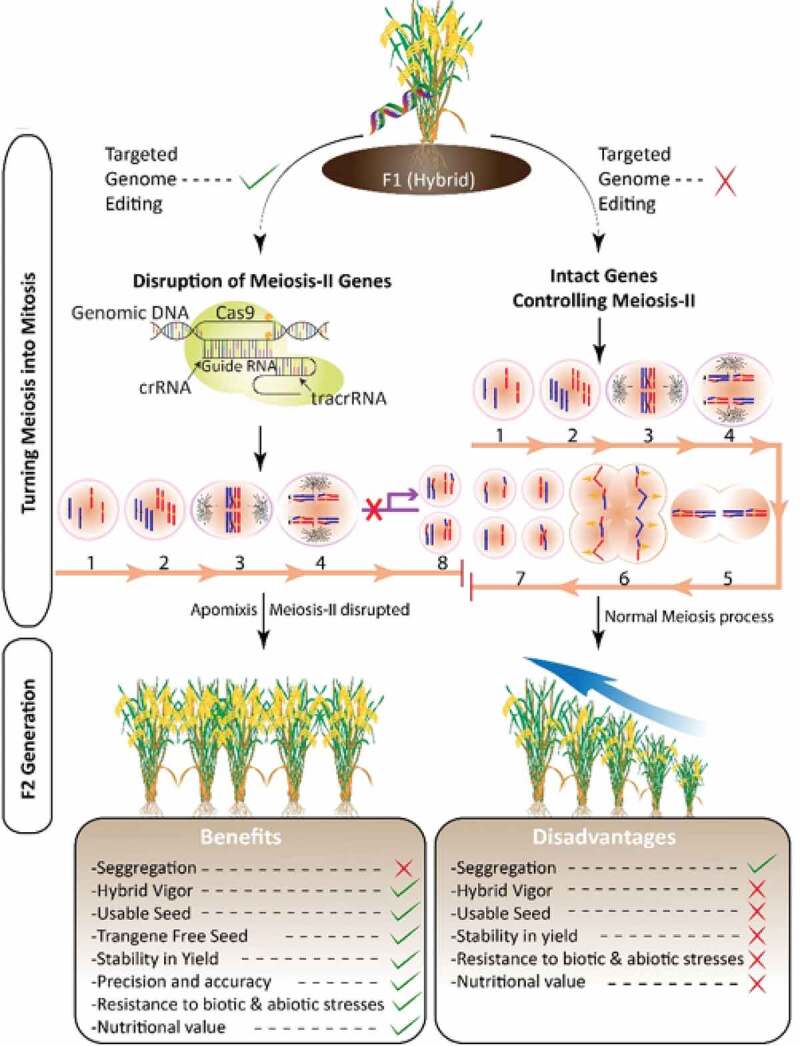
An illustration of promoting apomixis for hybrid vigor preservation through the CRISPR/Cas9 system. To fix hybrid vigor in F2 scientist are promoting apomixis in hybrid seed to keep intact the characteristics of F1 hybrid seed. The MiMe phenotypes is achieved through disruption of gene-controlling meiosis II which leads toward unreduced embryo sac essential for apomeiosis. However, in other case the hybrid segregate in F2 plant populations and all the important characteristics are lost. 1, Interphase; 2, Prophase-I; 3, Metaphase-I; 4, Anaphase-I; 5, Metaphase-II; 6, Anaphase-II; 7, n gametes; 8, 2 n gametes; NBTs, (new breeding techniques); P/TGMS, Cas9; CRISPR- associated protein 9, tracrRNA (trans-activating RNA).

The only drawback to hybrid technology includes the loss of hybrid vigor along with other desired traits due to genetic segregations in subsequent generations. Moreover, the cost of new seeds for every sowing is a major challenge faced by subsistence farmers.^78^ The preservation of hybrid seed qualities has significant implications for ensuring food security, environmental preservation, and employment. Ensuring that crops pass on hybrid qualities to seeds has been a major challenge, but researchers equipped with modern plant breeding technologies are striving to overcome this challenge. The findings in the abovementioned studies revealed a straightforward strategy to promote apomixis to preserve hybrid vigor in rice. Moreover, this approach can also work for other cereal crops that have equivalent genes, such as wheat, corn, barley, and millet, by increasing the percentage of seeds with the same hybrid vigor, and ultimately, these seeds can reach farmers’ fields. Moreover, identifying genes controlling the molecular mechanism of apomictic reproduction is critical to understanding its genetic architecture, which is still little understood (Fig. 3).

## 7. Limitations to Synthetic Apomixis

The utilization of the CRISP/Cas system for synthetic apomixis has been successfully employed in rice; however, seed production with intact hybrid vigor was significantly reduced.,13reported that *MiMe* does not significantly reduce seed production, whereas a mutation in the *MTL* gene leads to haploid induction at the expense of seed production in rice. Similarly, in maize, large-scale chromosome fragmentation caused a reduction in fertility owing to post meiosis in the *mtl* gametophyte.^4^ Fragmentation leads to an imbalance between the genomes of both the maternal and paternal sides. Several genes involved in embryo and endosperm development have been investigated in *Arabidopsis* and apomictic plants. A novel strategy, i.e., combining the *Fix* (*Fixation of hybrids*) strategy with genes promoting autoendosperm development, may resolve the issue of low fertility caused by the *Fix* strategy. In the *Fix* strategy, clonal diploid gametes are produced, similar to the *MiMe* strategy. After self-fertilization, in addition to tetraploid hybrids, clonal diploid hybrids are produced in the offspring. Using the *Fix* strategy, we successfully introduced synthetic apomixis into hybrid rice. However, seed production, which is one of the most important traits for rice cultivation, was significantly reduced in both *Fix* and clonal *Fix* plants. Therefore, the *Fix* strategy can be successfully employed in crops where seed production is a secondary objective, e.g., vegetables and forage crops, but this also needs further investigation.,9reported that the engineered *Fix* strategy was not as effective as naturally occurring apomixis because of penetration ability. Therefore, further studies, such as the identification of new parthenogenesis-inducing genes, are required to determine how to increase the frequency of clonal seeds. In contrast, techniques to distinguish clonal seeds or plants among offspring could be developed to further expand the application of the *Fix* strategy in agriculture. The mechanism of heterosis is less known, and it is generally believed that the degree of genetic distance contributes to heterosis. ^79^ However, recent studies have shown that epigenetic involvement also contributes to heterosis, ^80,14^ re-sequenced the whole genome of parent plants and clonal plants and reported the reliable transmission of genetic information, which demonstrated that epigenetic information was also transferred normally across generations. Although epigenetic modification cannot be ruled out, the modifications were too minor to induce detectable phenotypic changes in rice under field conditions. Further studies are thus required in several other crops to investigate phenotypic stability over multiple generations.

## 8. Conclusion and Future Perspectives

Understanding the phenomenon of apomixis remains challenging for plant geneticists; however, its potential benefits remain a focus of enormous interest for plant breeders. Both naturally existing genes and induced mutations can divert the natural sexual pathway toward apomixis, which can be further investigated. A combined strategy that can target apomeiosis, parthenogenesis, and seed formation might be of the utmost importance to exploit the full potential of apomixis to eliminate breeding barriers. It has been historically proven that the development of hybrids for different crops has helped farmers obtain benefits, such as maximum crop yields and grain quality as well as increased resistance to biotic and abiotic stresses, but hybrid seed lacks the ability to produce plants with the same qualities in subsequent generations. Hence, farmers have had no option other than to buy expensive hybrid seeds every year. The identification of QTLs/genes through new mapping technologies, that is, comparative mapping, linkage disequilibrium mapping, deletion mapping, and new high-throughput sequencing methods, will help to reveal core apomixis chromosomal regions. Similarly, high-throughput technologies can expose or remove the evolutionary genetic load of genes and epigenetic modifications related to apomixis and can be further utilized in agriculture as a tool to fix elite genotypes that are important for food security. In summary, the successful application of genome editing technology for targeted mutagenesis has opened further avenues of research for understanding the molecular mechanisms controlling apomixis. The efficiency of clonal propagation in crops, particularly in rice, has been limited by the frequency of parthenogenesis. However, this could be improved in the future by studying different promoters of genes or incorporating specific alleles that exhibit full or partial dominance.
